# Supplementary material for: Analysis of PPARγ Signaling Activity in Psoriasis
Source: Int J Mol Sci. 2021 Aug 10;22(16):8603. doi: 10.3390/ijms22168603 (PMC8395241; doi:10.3390/ijms22168603)
Supplement: Supplementary file 1 [file ijms-22-08603-s001.zip › Supplemental materials_Analysis of PPARg signaling activity in psoriasis/Pathway models/Models images and html files/Model of PPARG related pathways in psoriasis (short version)/11683.html]

TSC2


# Protein TSC2

|  |  |
| --- | --- |
| URN | urn:agi-llid:7249 |
| Total Entities | 0 |
| Connectivity | 2180 |
| Name | TSC2 |
| Description | tuberous sclerosis 2 |
| Notes | Mutations in this gene lead to tuberous sclerosis complex. Its gene product is believed to be a tumor suppressor and is able to stimulate specific GTPases. The protein associates with hamartin in a cytosolic complex, possibly acting as a chaperone for hamartin. Alternative splicing results in multiple transcript variants encoding different isoforms. [provided by RefSeq, Jul 2008] |
| ObjectType | Protein |

---

|  |  |
| --- | --- |
| Pathway | Oncogene Induced Cellular Senescence |
|  | DNA Persistent Repair Inhibits mTOR Signaling |
|  | mTOR Signaling Activation by Fatty Acids and Glucose |
|  | mTOR Signaling Activation by Amino Acids |
|  | mTOR Signaling |
|  | mTOR Signaling Hyperfunction |
|  | Hypoxia Inhibits mTOR Signaling |
|  | SIRT1 Signaling in Aging |
|  | Insulin Influence on Protein Synthesis |
|  | Insulin Signaling |
|  | Preconditioning Ischemia |
|  | Proteins Involved in Diabetes Mellitus Type 2 |
|  | Glucocorticoids in Insulin Resistance in Skeletal Muscles |
|  | Proteins Involved in non-Alcoholic Fatty Liver Disease |
|  | Peutz-Jeghers Syndrome |
|  | Proteins Involved in Peutz-Jeghers Syndrome |
|  | Proteins Involved in Pancreatic Cancer |
|  | Proteins Involved in Epilepsy |
|  | Ketogenic Diet Mechanism (Hypothesis) |
|  | Blood-Brain Barrier Disruption in Epileptiform Disorders |
|  | mTOR Hyperactivation after Status Epilepticus |
|  | Angiogenesis in Endometriosis |
|  | Androgen Receptor/SGK1 Signaling |
|  | Polycystins Mutation Effects in Cystic Kidney Disease |
|  | Proteins Involved in Polycystic Kidney Disease |
|  | AREG -> HIF1A Expression Targets |
|  | EGF -> HIF1A Expression Targets |
|  | EGFR/ERBB2 -> HIF1A Signaling |
|  | EREG -> HIF1A Expression Target |
|  | HBEGF -> HIF1A Expression Targets |
|  | N2 Neutrophils in Tumor-Promoting Inflammation and Tumor Progression |
|  | TGFB Signaling activation by Blocking of Tumor Suppressors |
|  | PI3K/AKT/MTOR Signaling Activation by Blocking of Tumor Suppressors |
|  | HIF1A as Master Regulator of Angiogenesis in Cancer |
|  | MTOR/TORC in Cancer Cell Motility and Invasion |
|  | TGFB Family in Epithelial to Mesenchymal Transition in Cancer |
|  | Metabolic Effects of Oncogenes and Tumor Suppressor in Cancer Cells |
|  | Genes with Mutations in Cancer Metabolic Reprogramming |
|  | Proteins with Altered Expression in Cancer Metabolic Reprogramming |
|  | NRG1 -> HIF1A Expression Target |
|  | Androgen Receptor non-Genomic Signaling |
|  | TGF-beta Signaling |
|  | AMPK Signaling |
|  | mTOR Signaling |
|  | VEGF Signaling |
|  | EGFR Signaling |
|  | TGFA -> HIF1A Expression Targets |
|  | Model of PPARG signaling in psoriasis |
|  | PPARG negative regulators and targets |
|  | Model of PPARG related pathways in psoriasis (short version) |
|  | 1\_Formation of cysts in polycystic kidney disease |
|  | 3\_Enhanced angiogenesis in endometriosis |
|  | 2\_Neuron and Schwann cell death |
|  | Model of PPARG signaling in psoriais (tested) |

---

|  |  |
| --- | --- |
| Group | Genes with Mutations Associated with Pancreatic Cancer |
|  | Proteins Involved in Cowden Syndrome |
|  | Genes Associated with Syndromic Epilepsy |
|  | Genes Associated with Mental Retardation and Brain Malformations in Epilepsy |

---

|  |  |
| --- | --- |
| MedScan ID | 7249 |

---

|  |  |
| --- | --- |
| LocusLink ID | 7249 |
|  | 22084 |
|  | 24855 |

---

|  |  |
| --- | --- |
| Alias | tuberin isoform III |
|  | Tuberins |
|  | tuberin isoform II |
|  | tuberous sclerosis complex 2 protein |
|  | tuberous sclerosis-II |
|  | PPP1R160 |
|  | FLJ43106 |
|  | OTTHUMP00000198394 |
|  | tuberous sclerosis 2 |
|  | Tuberous sclerosis 2, (renal carcinoma) |
|  | tumor suppressor protein tuberous sclerosis complex 2 |
|  | TSC complex subunit 2 |
|  | tuberin isoform I |
|  | tuberin isoform 2 |
|  | TCS2 |
|  | TSC4 |
|  | tuberin isoform 3 |
|  | LOC124054 |
|  | Tuberin |
|  | tuberous sclerosis-2 |
|  | tuberin isoform 1 |
|  | protein phosphatase 1, regulatory subunit 160 |
|  | tuberous sclerosis 2 homolog protein |
|  | tuberous sclerosis II |
|  | TSC2 |
|  | LAM |
|  | tuberous sclerosis 2 protein |
|  | Nafld |
|  | tuberous sclerosis 2 protein homolog |
|  | Rc |
|  | renal carcinoma |

---

|  |  |
| --- | --- |
| GO ID | 0005096 |
|  | 0051879 |
|  | 0019902 |
|  | 0042803 |
|  | 0031267 |
|  | 0043276 |
|  | 0006897 |
|  | 0007507 |
|  | 0048009 |
|  | 0032007 |
|  | 0030178 |
|  | 0008285 |
|  | 0046627 |
|  | 1901525 |
|  | 0014067 |
|  | 0051898 |
|  | 0006469 |
|  | 0001843 |
|  | 0050918 |
|  | 0043547 |
|  | 0016239 |
|  | 0006606 |
|  | 0043491 |
|  | 0008104 |
|  | 0051726 |
|  | 0030100 |
|  | 0046626 |
|  | 0051056 |
|  | 0016192 |
|  | 0016032 |
|  | 0005794 |
|  | 0033596 |
|  | 0005737 |
|  | 0005829 |
|  | 0005764 |
|  | 0016020 |
|  | 0005634 |
|  | 0048471 |
|  | 0014069 |
|  | 0071889 |
|  | 0047485 |
|  | 0046982 |
|  | 0007568 |
|  | 0030030 |
|  | 0032869 |
|  | 0030010 |
|  | 0045184 |
|  | 0046323 |
|  | 0043407 |
|  | 1904262 |
|  | 0050771 |
|  | 0045792 |
|  | 0050680 |
|  | 0010719 |
|  | 0048147 |
|  | 0016242 |
|  | 0048550 |
|  | 0001933 |
|  | 1905563 |
|  | 0014065 |
|  | 0010508 |
|  | 0045785 |
|  | 0060999 |
|  | 0010763 |
|  | 0010976 |
|  | 0045944 |
|  | 0051291 |
|  | 0051260 |
|  | 0034394 |
|  | 0044861 |
|  | 0099175 |
|  | 0001666 |
|  | 0007165 |
|  | 0035176 |
|  | 0005901 |
|  | 0042995 |
|  | 0005856 |
|  | 0030425 |
|  | 0098978 |
|  | 0030426 |
|  | 0043231 |
|  | 0045121 |
|  | 0043005 |
|  | 0043025 |
|  | 0032991 |
|  | 0045202 |
|  | 0007050 |
|  | 0071500 |
|  | 0043234 |
|  | 0038095 |
|  | 0006953 |
|  | 0007173 |
|  | 0008543 |
|  | 0045087 |
|  | 0008286 |
|  | 0048011 |
|  | 0048015 |
|  | 0032320 |
|  | 0005623 |
|  | 0005622 |
|  | 0005097 |
|  | 0032851 |

---

|  |  |
| --- | --- |
| KEGG ID | hsa:7249 |
|  | rno:24855 |
|  | mmu:22084 |

---

|  |  |
| --- | --- |
| Organism | Homo sapiens {Organism urn:agi-taxid:9606} |
|  | Mus musculus {Organism urn:agi-taxid:10090} |
|  | Rattus norvegicus {Organism urn:agi-taxid:10116} |
|  | Homo sapiens |
|  | Mus musculus |
|  | Rattus norvegicus |

---

|  |  |
| --- | --- |
| Mouse chromosome position | 17 12.41 cM |

---

|  |  |
| --- | --- |
| OMIM ID | 191092 |
|  | 607341 |
|  | 606690 |
|  | 613254 |

---

|  |  |
| --- | --- |
| Rat chromosome position | 10q12 |

---

|  |  |
| --- | --- |
| Hugo ID | 12363 |
|  | HGNC:12363 |

---

|  |  |
| --- | --- |
| Human chromosome position | 16p13.3 |

---

|  |  |
| --- | --- |
| Swiss-Prot Accession | P49815 |
|  | X5D7Q2 |
|  | B3KWH7 |
|  | Q5HYF7 |
|  | H3BMQ0 |
|  | P49815.2 |
|  | Q3UGI8 |
|  | Q7TT21 |
|  | Q3UHB2 |
|  | Q3UG88 |
|  | Q3TQ10 |
|  | Q61037.1 |
|  | P49816 |
|  | A0A0G2JSL4 |
|  | P49816.1 |
|  | A7E2E2 |
|  | B4DIL8 |
|  | B4DIQ7 |
|  | B4DRN2 |
|  | B7Z2B8 |
|  | C9J378 |
|  | O75275 |
|  | Q4LE71 |
|  | Q8TAZ1 |
|  | X5D2U8 |
|  | Q61037 |

---

|  |  |
| --- | --- |
| PIR ID | A49420 |
|  | S57329 |

---

|  |  |
| --- | --- |
| GenBank ID | NC\_000016 |
|  | XM\_011522638 |
|  | XP\_011520940 |
|  | XM\_017023617 |
|  | XP\_016879106 |
|  | NM\_001114382 |
|  | NP\_001107854 |
|  | NM\_001363528 |
|  | NP\_001350457 |
|  | NM\_001077183 |
|  | NP\_001070651 |
|  | NM\_001318827 |
|  | NP\_001305756 |
|  | NM\_001318831 |
|  | NP\_001305760 |
|  | NM\_001318829 |
|  | NP\_001305758 |
|  | NM\_000548 |
|  | NP\_000539 |
|  | NM\_001370405 |
|  | NP\_001357334 |
|  | NM\_001370404 |
|  | NP\_001357333 |
|  | NM\_021055 |
|  | NP\_066399 |
|  | XM\_017023618 |
|  | XP\_016879107 |
|  | XM\_011522636 |
|  | XP\_011520938 |
|  | XM\_011522637 |
|  | XP\_011520939 |
|  | XM\_017023615 |
|  | XP\_016879104 |
|  | XM\_011522639 |
|  | XP\_011520941 |
|  | XM\_011522640 |
|  | XP\_011520942 |
|  | XM\_024450413 |
|  | XP\_024306181 |
|  | NM\_001318832 |
|  | NP\_001305761 |
|  | NG\_005895 |
|  | AB014460 |
|  | BAA32694 |
|  | AC005600 |
|  | AAC34210 |
|  | AC093513 |
|  | AH004937 |
|  | AAB41564 |
|  | CH471112 |
|  | EAW85556 |
|  | EAW85557 |
|  | EAW85558 |
|  | EAW85559 |
|  | EAW85560 |
|  | KY922729 |
|  | AWN00470 |
|  | MG589762 |
|  | AYE20492 |
|  | AB210000 |
|  | BAE06082 |
|  | AK022401 |
|  | AK094152 |
|  | BAG52831 |
|  | AK125096 |
|  | BAG54139 |
|  | AK294548 |
|  | BAH11804 |
|  | AK295672 |
|  | BAG58530 |
|  | AK295728 |
|  | BAG58569 |
|  | AK299343 |
|  | BAG61344 |
|  | BC025364 |
|  | AAH25364 |
|  | BC046929 |
|  | AAH46929 |
|  | BC150300 |
|  | AAI50301 |
|  | BF194994 |
|  | BM966762 |
|  | BX647816 |
|  | CAI46010 |
|  | CA427637 |
|  | DA316794 |
|  | DB288276 |
|  | DC402106 |
|  | KJ534954 |
|  | AHW56594 |
|  | KJ534955 |
|  | AHW56595 |
|  | KJ534956 |
|  | AHW56596 |
|  | KJ534957 |
|  | AHW56597 |
|  | KJ534958 |
|  | AHW56598 |
|  | KJ534982 |
|  | AHW56622 |
|  | KJ534983 |
|  | AHW56623 |
|  | KJ534984 |
|  | AHW56624 |
|  | KJ534985 |
|  | AHW56625 |
|  | KJ535011 |
|  | AHW56650 |
|  | KJ535022 |
|  | AHW56661 |
|  | KJ535038 |
|  | AHW56677 |
|  | KJ535039 |
|  | AHW56678 |
|  | KJ535051 |
|  | AHW56690 |
|  | KJ535091 |
|  | AHW56730 |
|  | KJ535094 |
|  | AHW56733 |
|  | LC224282 |
|  | BAX02962 |
|  | LC224283 |
|  | BAX02963 |
|  | LC224284 |
|  | BAX02964 |
|  | LC224285 |
|  | BAX02965 |
|  | LC224286 |
|  | BAX02966 |
|  | LC224287 |
|  | BAX02967 |
|  | LC224288 |
|  | BAX02968 |
|  | LC224289 |
|  | BAX02969 |
|  | LC224290 |
|  | BAX02970 |
|  | LC224291 |
|  | BAX02971 |
|  | LC224292 |
|  | BAX02972 |
|  | LC224293 |
|  | BAX02973 |
|  | LC224294 |
|  | BAX02974 |
|  | S82474 |
|  | AAB37376 |
|  | X75621 |
|  | CAA53287 |
|  | P49815 |
|  | NC\_000083 |
|  | NM\_001286716 |
|  | NP\_001273645 |
|  | NM\_001286714 |
|  | NP\_001273643 |
|  | NM\_001286718 |
|  | NP\_001273647 |
|  | NM\_001039363 |
|  | NP\_001034452 |
|  | NM\_011647 |
|  | NP\_035777 |
|  | NM\_001286720 |
|  | NP\_001273649 |
|  | NM\_001286713 |
|  | NP\_001273642 |
|  | XM\_017317392 |
|  | XP\_017172881 |
|  | XM\_017317389 |
|  | XP\_017172878 |
|  | XM\_017317388 |
|  | XP\_017172877 |
|  | XM\_017317387 |
|  | XP\_017172876 |
|  | XM\_017317385 |
|  | XP\_017172874 |
|  | XM\_006524044 |
|  | XP\_006524107 |
|  | XM\_006524040 |
|  | XP\_006524103 |
|  | XM\_017317386 |
|  | XP\_017172875 |
|  | XM\_017317384 |
|  | XP\_017172873 |
|  | XM\_017317390 |
|  | XP\_017172879 |
|  | XM\_017317393 |
|  | XP\_017172882 |
|  | XM\_017317391 |
|  | XP\_017172880 |
|  | XR\_001782062 |
|  | XR\_001782063 |
|  | AB009371 |
|  | BAA28845 |
|  | AC132367 |
|  | AF333927 |
|  | AAK48834 |
|  | CH466606 |
|  | EDL22341 |
|  | EDL22342 |
|  | EDL22343 |
|  | EDL22344 |
|  | EDL22345 |
|  | U38921 |
|  | AF132986 |
|  | AAD27867 |
|  | AK143799 |
|  | AK147016 |
|  | BAE27610 |
|  | AK147302 |
|  | AK147487 |
|  | BAE27945 |
|  | AK147874 |
|  | AK147908 |
|  | BAE28220 |
|  | AK148062 |
|  | BAE28321 |
|  | AK148072 |
|  | BAE28325 |
|  | AK148271 |
|  | AK163991 |
|  | BAE37575 |
|  | AK164586 |
|  | BAE37839 |
|  | AK170589 |
|  | BAE41898 |
|  | AK171007 |
|  | BAE42178 |
|  | AK177552 |
|  | AK178821 |
|  | AK179783 |
|  | AK179817 |
|  | AK181602 |
|  | AK182281 |
|  | AK182662 |
|  | AK182995 |
|  | AK183282 |
|  | AK183464 |
|  | AK183501 |
|  | AK183941 |
|  | AK184422 |
|  | AK184645 |
|  | AK184680 |
|  | AK184991 |
|  | AK185522 |
|  | AK186134 |
|  | AK187103 |
|  | AK187131 |
|  | AK187383 |
|  | AK188248 |
|  | AK190188 |
|  | AK190229 |
|  | AK191260 |
|  | AK191520 |
|  | AK191587 |
|  | AK193509 |
|  | AK196271 |
|  | AK197601 |
|  | AK198541 |
|  | AK198980 |
|  | AK199927 |
|  | AK200853 |
|  | AK201826 |
|  | AK202101 |
|  | AK202630 |
|  | AK203056 |
|  | AK203435 |
|  | AK204110 |
|  | AK204112 |
|  | AK205325 |
|  | AK205469 |
|  | AK205980 |
|  | AK206076 |
|  | AK208040 |
|  | AK209880 |
|  | AK210945 |
|  | AK211775 |
|  | AK214850 |
|  | AK215427 |
|  | AK215802 |
|  | AK215985 |
|  | AK216627 |
|  | AK217069 |
|  | AK217739 |
|  | AK217801 |
|  | AK218459 |
|  | AK218943 |
|  | AK218975 |
|  | AK219279 |
|  | AK220072 |
|  | BC052207 |
|  | BC052449 |
|  | AAH52449 |
|  | BC060701 |
|  | AAH60701 |
|  | BY742714 |
|  | U37775 |
|  | AAA86901 |
|  | AAA86902 |
|  | U39818 |
|  | AAB18754 |
|  | Q61037 |
|  | NC\_005109 |
|  | NM\_012680 |
|  | NP\_036812 |
|  | XM\_006245909 |
|  | XP\_006245971 |
|  | XM\_017597022 |
|  | XP\_017452511 |
|  | XM\_017597020 |
|  | XP\_017452509 |
|  | XM\_017597019 |
|  | XP\_017452508 |
|  | XM\_017597027 |
|  | XP\_017452516 |
|  | XM\_017597026 |
|  | XP\_017452515 |
|  | XM\_017597024 |
|  | XP\_017452513 |
|  | XM\_017597025 |
|  | XP\_017452514 |
|  | XM\_017597023 |
|  | XP\_017452512 |
|  | XM\_017597021 |
|  | XP\_017452510 |
|  | XM\_008767544 |
|  | XP\_008765766 |
|  | AC\_000078 |
|  | AAHX01062902 |
|  | AAHX01062903 |
|  | AC093937 |
|  | CH473948 |
|  | EDM03846 |
|  | EDM03847 |
|  | EDM03848 |
|  | D84251 |
|  | BAA25108 |
|  | D85767 |
|  | D50413 |
|  | BAA08914 |
|  | U24150 |
|  | AAC52289 |
|  | P49816 |
|  | XM\_011522641 |
|  | XP\_011520943 |
|  | XM\_005255529 |
|  | XP\_005255586 |
|  | XM\_005255531 |
|  | XP\_005255588 |
|  | NC\_018927 |
|  | AMYH02031242 |
|  | L48517 |
|  | L48518 |
|  | L48519 |
|  | L48520 |
|  | L48522 |
|  | L48523 |
|  | L48524 |
|  | L48525 |
|  | L48526 |
|  | L48528 |
|  | L48529 |
|  | L48531 |
|  | L48535 |
|  | L48536 |
|  | L48537 |
|  | L48538 |
|  | L48539 |
|  | L48540 |
|  | L48541 |
|  | L48542 |
|  | L48543 |
|  | L48544 |
|  | L48546 |
|  | XM\_011246370 |
|  | XP\_011244672 |
|  | XM\_011246369 |
|  | XP\_011244671 |
|  | XM\_011246365 |
|  | XP\_011244667 |
|  | XM\_011246368 |
|  | XP\_011244670 |
|  | XR\_876467 |
|  | XM\_011246373 |
|  | XP\_011244675 |
|  | XM\_011246367 |
|  | XP\_011244669 |
|  | XM\_011246364 |
|  | XP\_011244666 |
|  | XM\_011246371 |
|  | XP\_011244673 |
|  | XM\_011246375 |
|  | XP\_011244677 |
|  | XM\_011246372 |
|  | XP\_011244674 |
|  | AC\_000039 |
|  | AAHY01131651 |
|  | AAHY01131652 |
|  | XM\_006245917 |
|  | XP\_006245979 |
|  | XM\_006245912 |
|  | XP\_006245974 |
|  | XM\_008767543 |
|  | XP\_008765765 |
|  | XM\_006245916 |
|  | XP\_006245978 |
|  | XM\_006245914 |
|  | XP\_006245976 |
|  | XM\_006245915 |
|  | XP\_006245977 |
|  | XM\_006245913 |
|  | XP\_006245975 |
|  | XM\_006245910 |
|  | XP\_006245972 |
|  | XM\_006245911 |
|  | XP\_006245973 |
|  | XM\_005255527 |
|  | XP\_005255584 |
|  | XM\_005255528 |
|  | XP\_005255585 |
|  | XM\_005255530 |
|  | XP\_005255587 |
|  | XM\_005255532 |
|  | XP\_005255589 |
|  | AC\_000148 |
|  | ABBA01030275 |
|  | ABBA01030276 |
|  | ABBA01030277 |
|  | ABBA01030278 |
|  | ABBA01030279 |
|  | XM\_006524041 |
|  | XP\_006524104 |
|  | XM\_006524038 |
|  | XP\_006524101 |
|  | XM\_006524045 |
|  | XP\_006524108 |
|  | XM\_006524039 |
|  | XP\_006524102 |
|  | XM\_006524037 |
|  | XP\_006524100 |
|  | XM\_006524042 |
|  | XP\_006524105 |
|  | XM\_006524046 |
|  | XP\_006524109 |
|  | XM\_006524043 |
|  | XP\_006524106 |
|  | XM\_005255533 |
|  | XP\_005255590 |
|  | XR\_243299 |
|  | XM\_005255534 |
|  | XP\_005255591 |
|  | AABR06063007 |

---

|  |  |
| --- | --- |
| Swiss-Prot ID | TSC2\_HUMAN |
|  | TSC2\_RAT |

---

|  |  |
| --- | --- |
| Cell Localization | Cytoplasm |
|  | Membrane |
|  | Nucleus |

---

|  |  |
| --- | --- |
| Ensembl ID | ENSG00000103197 |
|  | ENSP00000219476.3 |
|  | ENST00000219476.9 |
|  | ENSP00000384468.2 |
|  | ENST00000401874.7 |
|  | ENSP00000344383.4 |
|  | ENST00000350773.9 |
|  | ENSP00000399232.2 |
|  | ENST00000439673.6 |
|  | ENSP00000371978.6 |
|  | ENST00000382538.10 |
|  | ENSP00000454487.1 |
|  | ENST00000568454.6 |
|  | ENSP00000495459.1 |
|  | ENST00000642365.1 |
|  | ENSP00000496611.1 |
|  | ENST00000644329.1 |
|  | ENSMUSG00000002496 |
|  | ENSMUSP00000094986.1 |
|  | ENSMUST00000097373.1 |
|  | ENSMUSP00000154338.1 |
|  | ENSMUST00000227745.1 |
|  | ENSMUSP00000153792.1 |
|  | ENSMUST00000226398.1 |
|  | ENSMUSP00000154706.1 |
|  | ENSMUST00000228412.1 |
|  | ENSMUSP00000153869.1 |
|  | ENSMUST00000226284.1 |
|  | ENSMUSP00000153758.1 |
|  | ENSMUST00000227607.1 |
|  | ENSRNOG00000011375 |
|  | ENSRNOP00000016221.1 |
|  | ENSRNOT00000016221.3 |
|  | ENSRNOP00000039381.5 |
|  | ENSRNOT00000052138.6 |
|  | ENSP00000219476 |
|  | ENST00000219476 |
|  | ENSP00000384468 |
|  | ENST00000401874 |
|  | ENSP00000344383 |
|  | ENST00000350773 |
|  | ENSP00000399232 |
|  | ENST00000439673 |
|  | ENSP00000371978 |
|  | ENST00000382538 |
|  | ENSMUSP00000094986 |
|  | ENSMUST00000097373 |
|  | ENSRNOP00000016221 |
|  | ENSRNOT00000016221 |

---

|  |  |
| --- | --- |
| MGI ID | MGI:102548 |
|  | 102548 |

---

|  |  |
| --- | --- |
| RGD ID | 3908 |

---

|  |  |
| --- | --- |
| Unigene ID | Hs.90303 |
|  | Mm.30435 |
|  | Rn.5875 |

---

|  |  |
| --- | --- |
| Homologene ID | 462 |

---
